# Histone deacetylase 2 is essential for LPS‐induced inflammatory responses in macrophages

**DOI:** 10.1111/imcb.12203

**Published:** 2018-10-31

**Authors:** Chenming Wu, Ang Li, Jian Hu, Jiuhong Kang

**Affiliations:** ^1^ Clinical and Translational Research Center of Shanghai First Maternity & Infant Health Hospital Shanghai Key Laboratory of Signaling and Disease Research School of Life Science and Technology Tongji University Shanghai 200092 China; ^2^ Research Center for Translational Medicine East Hospital Tongji University School of Medicine Shanghai 200120 China; ^3^ Key Laboratory of Arrhythmias of the Ministry of Education of China East Hospital Tongji University School of Medicine Shanghai 200120 China

**Keywords:** Activation protein‐1, nuclear receptor corepressor, proinflammatory genes

## Abstract

The role of specific histone deacetylase (HDAC) proteins in regulating the lipopolysaccharide (LPS)‐induced inflammatory response and its underlying mechanisms are unclear. Here, HDAC2, a class I HDAC family protein, is essential for the LPS‐triggered inflammatory response in macrophages. LPS stimulation increases HDAC2 expression in macrophages. Knockdown of HDAC2 decreases the expression of proinflammatory genes, such as IL‐12, TNF‐α and iNOS following stimulation with LPS. The adoptive transfer of HDAC2 knockdown macrophages attenuates the LPS‐triggered innate inflammatory response *in vivo*, and these mice are less sensitive to endotoxin shock and *Escherichia coli*‐induced sepsis. Mechanistically, the c‐Jun protein is the main target of HDAC2‐mediated LPS‐induced production of proinflammatory cytokines. Moreover, HDAC2 knockdown increases the expression of c‐Jun, which directly binds the promoters of proinflammatory genes and forms nuclear receptor corepressor complexes to inhibit the transcription of proinflammatory genes in macrophages. These effects are rescued by c‐Jun expression. According to the chromatin immunoprecipitation analysis, HDAC2 also selectively suppresses c‐Jun expression by directly binding to its promoter and modifying histone acetylation after LPS stimulation. Our findings define a new function and mechanism of the HDAC2/c‐Jun signaling network that regulates the LPS‐induced immune response in macrophages.

## Graphical abstract

Histone deacetylase HDAC2, a class I HDAC, is essential for LPS‐induced proinflammatory genes expression. HDAC2‐knockdown macrophages attenuated the LPS‐triggered innate inflammatory response, and these mice transferred with HDAC2‐knockdown macrophages were resistant to endotoxin shock.

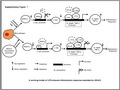

## Introduction

During gram‐negative bacteria‐induced sepsis, lipopolysaccharide (LPS) drives the systemic production of inflammatory genes, such as interleukin (IL)‐1, IL‐6, IL‐8 and tumor necrosis factor‐alpha (TNF‐α), which all induce sepsis‐associated pathology. Toll‐like receptor 4 (TLR4), a key member of the TLR family of pattern recognition receptors, is used by cells of the innate immune system to initiate immune responses upon recognition of LPS.^1^, ^2^ TLR4 initiates distinct and shared signaling pathways, depending on the adapters TRIF and/or MyD88, thereby inducing the activation of transcription factors, such as activator protein 1 (AP‐1), interferon regulatory factor 3 (IRF3), IRF7 and/or NF‐κB, which subsequently increases the secretion of proinflammatory cytokines and the expression of antimicrobial genes.^3^, ^4^, ^5^, ^6^ After stimulation, IL‐1 receptor‐associated kinases are activated by MyD88, which subsequently activate mitogen‐activated protein kinases, IKK‐NF‐κB and IKK‐NF‐κB.^4^, ^7^, ^8^ TRIF activates IRF3, increasing the secretion of proinflammatory cytokines, which are important for the adaptive induction of CD8^+^T cells.^9^ Although the LPS‐TLR4 signaling pathways are well established, epigenetic changes and mechanisms underlying the regulation of LPS‐TLR4 signaling pathways remain unclear.

Epigenetic changes are well known to play critical roles in the inflammatory response in macrophages and include chromatin modification, chromatin remodeling and changes in adaptors that bridge chromatin‐bound transcription factors or local modification of histones through the transcriptional machinery.^10^, ^11^, ^12^, ^13^, ^14^, ^15^ Epigenetic changes mediate multiple cellular functions, such as cellular activation, cellular differentiation and cellular proliferation. Dynamic changes in histone modifications and DNA methylation are involved in altering gene expression.^16^, ^17^ Histone acetylation is widely accepted to be a crucial event in the epigenetic regulatory mechanism, control of chromatin structure, transcription factor DNA accessibility and gene expression.^18^ Histone acetylation has recently been reported to be induced in macrophages in response to TLR stimulation and plays important roles in regulating the expression of multiple proinflammatory cytokine genes.^15^, ^19^

Histone deacetylases (HDACs), a family of lysine deacetylases, target histones and a variety of non‐histone proteins.^20^ The classic HDAC family, whose components are all similarly sensitive to HDAC inhibitors (HDACis),^21^ includes class I (HDAC1, HDAC2, HDAC3 and HDAC8) and class II HDACs. Class I HDACs, which are nuclear enzymes, are constitutively expressed in virtually all cell types and play key roles in regulating the expression of inflammatory genes.^22^, ^23^ HDAC inhibitors have been shown to downregulate the expression of many host defense genes, including kinases, cytokines, pattern recognition receptors, chemokines, transcription regulators, co‐stimulatory molecules and growth factors.^24^ As shown in our previous study, class I HDAC inhibitors suppress LPS‐induced expression of proinflammatory genes in macrophages.^25^ However, the specific class I HDAC protein responsible for the global rearrangement of the histone acetylation during LPS‐induced innate immune response and its underlying molecular mechanisms remain unclear. Here, we reported a role for HDAC2 in determining the commitment to the LPS‐induced inflammatory response. HDAC2 expression was induced in macrophages stimulated with LPS. Additionally, the expression of proinflammatory genes and proteins was reduced in LPS‐activated macrophages upon HDAC2 knockdown with HDAC2‐specific short hairpin RNAs (shRNAs). Adoptive transfer of HDAC2 knockdown macrophages protected mice from LPS and *Escherichia coli* challenge *in vivo* and prolonged their survival. HDAC2 affected the LPS‐induced inflammatory response by directly binding to c‐Jun promoters and regulating c‐Jun expression, which are essential for the negative regulation of the inflammatory response. Based on these findings, HDAC2 is involved in the mechanism regulating the LPS‐triggered innate immune response.

## Results

### LPS stimulation of macrophages induces HDAC2 expression

**Figure 1:**
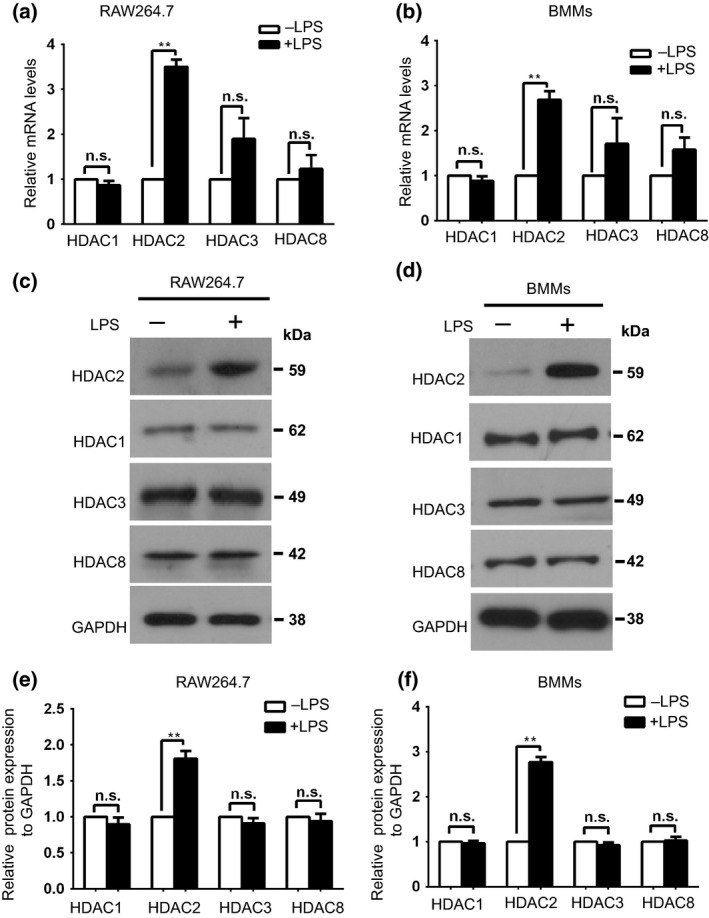
LPS stimulation of macrophages induces HDAC2 expression. **(a**,** b)** Levels of the mRNAs encoding HDAC2 and other class I HDAC isoforms in **(a)** RAW264.7 cells and **(b)** mouse BMMs after 1 h of stimulation with LPS (100 ng mL ^−1^). Levels of the HDAC1, HDAC2, HDAC3 and HDAC8 mRNAs were assessed using RT‐qPCR and normalized to untreated cells. Results are presented relative to untreated macrophage, which were set to 1. GAPDH was used as the internal control. Data are presented as the means ± s.d. of three independent experiments. n.s.: not significant, **P < 0.01 (two‐tailed Student's *t*‐test). **(c**,** d)** Half of the cells from **a**,** b** were lysed and western blots were performed with the indicated antibodies (HDAC1, HDAC2, HDAC3 and HDAC8). **(e)** Quantitative analysis of the western blots shown in **c. (f)** Quantitative analysis of the western blots shown in **d**. The data shown in **e** and **f** are presented as the means ± s.d. of three independent experiments, and results are presented relative to untreated macrophages, which were set to 1. n.s.: not significant, **P < 0.01 (two‐tailed Student's *t*‐test).

We investigated the expression of class I HDAC isoforms in macrophages after the LPS treatment to determine which specific class I HDAC isoforms play a vital role in the LPS‐triggered inflammatory response in macrophages. As shown in Figure 1a, b, HDAC2 expression was induced by LPS exposure. LPS treatment of the RAW264.7 cells and mouse primary bone‐marrow‐derived macrophages (BMMs) upregulated the expression of the HDAC2 mRNA within 1 h of stimulation. The level of the HDAC2 protein was also increased in the LPS‐stimulated cells compared with the unstimulated macrophages (Figure 1c–f). HDAC1, HDAC3 and HDAC8 levels, which are all considered class I HDACs, were not affected by LPS stimulation (Figure 1a–f). Thus, HDAC2, an epigenetic regulator, is part of the specific LPS‐induced macrophage activation signature.

### HDAC2 regulates proinflammatory cytokine production in LPS‐stimulated macrophages

**Figure 2:**
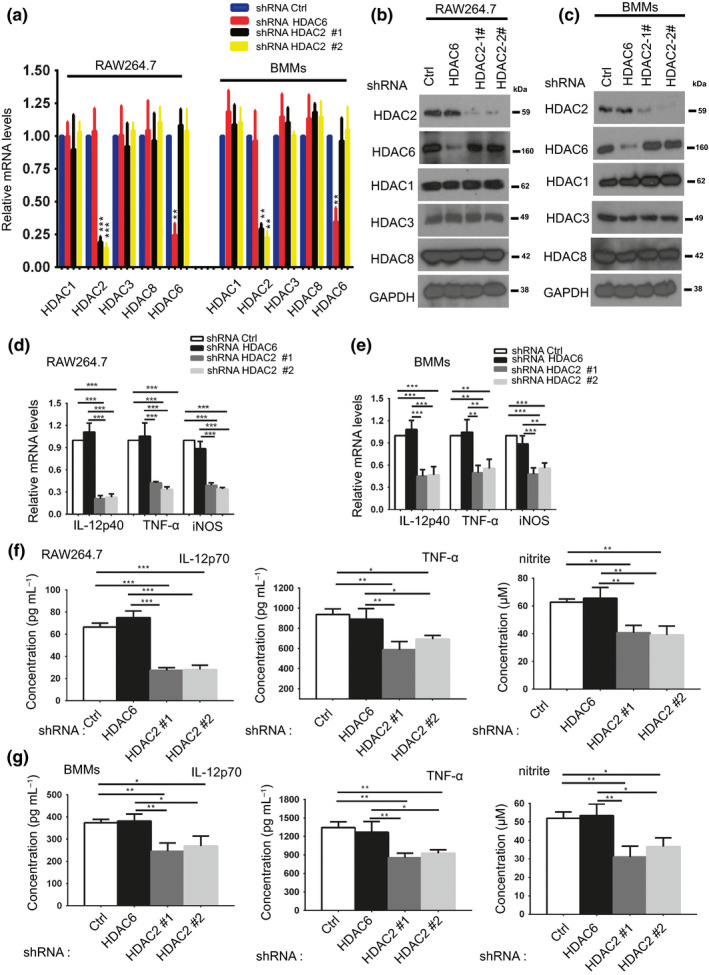
HDAC2 knockdown alters the expression of proinflammatory genes in macrophages. **(a)** RAW 264.7 cells or BMMs were infected with an HDAC2 shRNA lentivirus (shRNA HDAC2#1 and shRNA HDAC2#2) or control shRNA lentivirus (shRNA HDAC6 and shRNA Ctrl). Half of the cells were used to determine the mRNA levels of the target genes (HDAC1, HDAC2, HDAC3, HDAC8 and HDAC6) using RT‐qPCR. RT‐qPCR was employed to determine the knockdown efficiency. The data are presented relative to shRNA Ctrl macrophages, which were set to 1. GAPDH was used as the internal control. Data are presented as the means ± s.d. of three independent experiments. **P < 0.01, and ***P < 0.001 (two‐tailed Student's *t*‐test) compared with the shRNA Ctrl. **(b**,** c)** Half of the cells from **a** were lysed and western blots were performed with the indicated antibodies. Western blots were utilized to detect the knockdown efficiency. **(d**,** e)** Macrophages (RAW264.7 cells and BMMs) stably expressing Ctrl, HDAC6 or HDAC2 shRNAs shown in **a** were treated with LPS (100 ng mL ^−1^) for 6 h. Levels of the IL‐12p40, TNF‐α and iNOS mRNAs were determined using RT‐qPCR. **(f**,** g)** Macrophages (RAW264.7 cells and BMMs) stably expressing Ctrl, HDAC6 or HDAC2 shRNAs shown in **a** were treated with LPS (100 ng mL ^−1^) for 24 h. After 24 h, the supernatants were collected to determine the concentrations of IL‐12p70 and TNF‐α using ELISAs. Additionally, nitrite concentrations, a stable end product of NO production, were detected using the Griess reagent. The data shown in **d** and **e** are presented relative to shRNA Ctrl macrophages, which were set to 1. GAPDH was used as the internal control. Data in **d**–**g** are presented as the means ± s.d. of three independent experiments. *P < 0.05, **P < 0.01, and ***P < 0.001 (one‐way ANOVA).

Proinflammatory cytokines play important roles in LPS‐induced inflammatory responses in macrophages. We next explored whether HDAC2 regulates the expression of proinflammatory genes in LPS‐stimulated mouse macrophages. Mouse macrophages were stably infected with a lentivirus carrying two shRNAs variants of HDAC2 to exclude possible off‐target effects. Furthermore, we infected mouse macrophages with a lentivirus encoding an HDAC6 shRNA (HDAC6 knockdown macrophages), as HDAC6 expression is not altered by LPS (HDAC6 knockdown macrophages) and the scrambled shRNA (shRNA Ctrl macrophages) served as the control group. We confirmed specific HDAC2 silencing in RAW264.7 cells and BMMs by assessing the levels of HDAC2 and other class I HDAC mRNAs and proteins (Figure 2a–c). Mouse proinflammatory genes were monitored 6 h after LPS exposure using RT‐qPCR, and proinflammatory proteins were detected after 24 h using ELISAs. As shown in Figure 2d, e, knockdown of endogenous HDAC2 with HDAC2 shRNAs dramatically decreased the levels of the IL‐12p40, TNF‐α and iNOS mRNAs compared with the HDAC6 knockdown macrophages and shRNA Ctrl macrophages. The concentrations of the IL‐12p70 and TNF‐α proteins in the supernatant of the HDAC2 knockdown macrophages were also less than the HDAC6 knockdown macrophages and shRNA Ctrl macrophages (Figure 2f, g). Moreover, knockdown of HDAC2 in macrophages significantly decreased the production of nitric oxide catalyzed by iNOS in the presence of LPS, as determined by evaluating the nitrite (a nitric oxide metabolite) concentrations (Figure 2f, g). We also confirmed that the downregulation of proinflammatory gene expression in HDAC2 knockdown macrophages was not caused by HDAC2 shRNA‐induced cytotoxicity, as evidenced by the comparable cell viability between HDAC2 knockdown and shRNA Ctrl macrophages (Supplementary figure 1a–c).

Cells were transduced with a lentivirus carrying the full‐length HDAC2 cDNA and treated with LPS to further investigate the role of HDAC2 in proinflammatory gene expression in macrophages. HDAC2 overexpression in macrophages induced the expression of proinflammatory genes in response to LPS stimulation (Supplementary figure 2a–f). Based on these findings, HDAC2 is important for sustaining proinflammatory cytokine expression in LPS‐stimulated macrophages.

### Adoptive transfer of HDAC2 knockdown BMMs protects mice from LPS and bacterial toxicity *in vivo*

**Figure 3:**
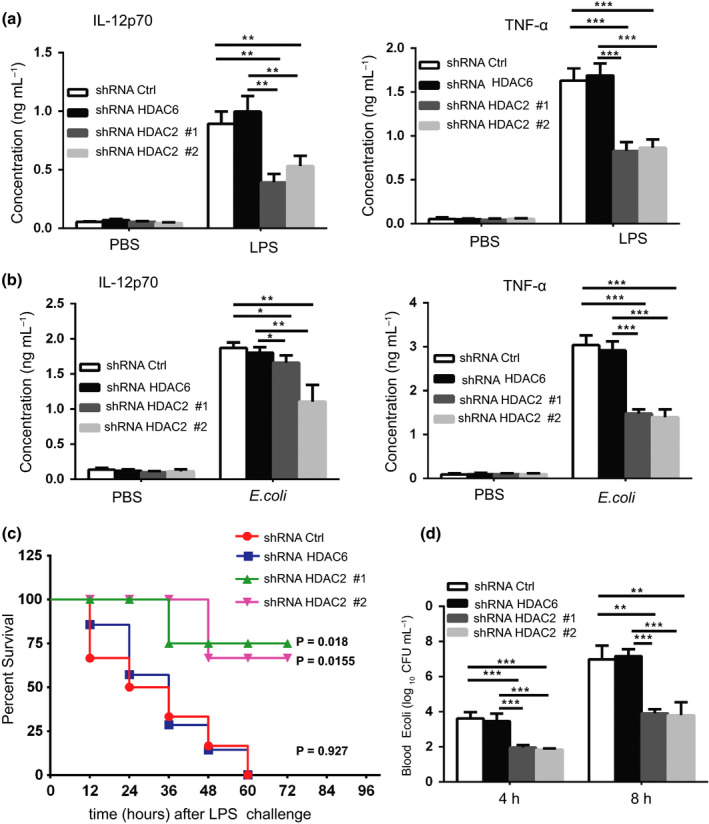
Adoptive transfer of HDAC2 knockdown BMMs protects mice from LPS challenge *in vivo*. Macrophages were depleted by a 10 mg kg^−1^ GdCl_3_ intravenous injection, and then 1 × 10^7^ HDAC2 knockdown (shRNA HDAC2#1 and shRNA HDAC2#2) or control (shRNA HDAC6 and shRNA Ctrl) BMMs were adoptively transferred into the mice via a tail vein injection, followed by challenge with LPS or *E. coli* infection. **(a**,** b)** Two hours later, serum TNF‐α and IL‐12p70 levels in the mice (*n* = 6 mice per genotype) transferred with various BMMs were determined using ELISAs. Data are presented from three independent experiments (means ± s.d.). **(c)** Survival of the mice (*n* = 6 mice per genotype) transferred with various BMMs (*n* = 6 mice per genotype) and subsequently administered LPS via an i.p. injection (15 mg kg^−1^ body weight). Data were compared with mice that received shRNA Ctrl BMMs (log‐rank (Mantel‐Cox) test). **(d)** Bacterial loads in the blood were also calculated. Each experimental group included six C57BL/6 mice. Means ± s.d. (error bars) of three independent experiments are shown. Significant differences in the data presented in **a**,** b** and **d** are designated as *P < 0.05, **P < 0.01, and ***P < 0.001 (one‐way ANOVA).

HDAC2 played a vital role in regulating LPS‐triggered proinflammatory gene expression *in vitro*. Thus, we hypothesized that the adoptive transfer of HDAC2 knockdown BMMs might affect the LPS‐induced innate immune response *in vivo*. We intravenously injected mice with GdCl_3_ to selectively deplete circulating mononuclear cells of the monocyte/macrophage lineage (Supplementary figure 3a),^13^, ^26^ and then adoptively transferred HDAC2 knockdown BMMs or control (HDAC6 knockdown and shRNA Ctrl) BMMs into the mice via tail vein injections, followed by LPS challenge. Mice with HDAC2 knockdown BMM transfer produced significantly less TNF‐α and IL‐12p70 than mice with control BMM transfer in response to LPS challenge (Figure 3a). HDAC2 knockdown or control BMM‐transferred mice were challenged with intact gram‐negative *E. coli* to further assess the effect of HDAC2 on the host macrophage‐mediated innate response to pathogen infection. After infection with *E*. *coli*, markedly reduced serum TNF‐α and IL‐12p70 levels were observed in HDAC2 knockdown BMM‐transferred mice compared with their control littermates (Figure 3b). In addition, mice that received adoptive transfer of the HDAC2 knockdown BMMs exhibited prolonged survival and were more resistant to endotoxic shock compared with mice transferred with the shRNA Crtl BMMs after lethal LPS challenge (Figure 3c). Accordingly, HDAC2 knockdown BMM‐transferred mice displayed less *E. coli* in the blood (Figure 3d). This result was consistent with published findings that proinflammatory cytokine production is associated with the bactericidal activity of macrophages.^27^ Thus, HDAC2 also plays a critical role in the LPS‐induced innate inflammatory response and endotoxic shock *in vivo*.

### C‐Jun is a key target of HDAC2 and regulates LPS‐triggered inflammatory response in macrophages

HDACs are important epigenetic regulators that control chromatin structure, transcription factor DNA accessibility and gene expression by altering the histone acetylation at the promoter regions of genes. Chromatin immunoprecipitation (ChIP) assays were performed to determine whether HDAC2 binds to the promoter regions of proinflammatory genes in macrophages treated with LPS to obtain additional insights into the potential chromatin modifications mediated by HDAC2. An obvious difference in the ability of HDAC2 to bind to the IL‐12p40, TNF‐α and iNOS promoters was not observed between LPS‐stimulated and unstimulated macrophages (Supplementary figure 4a, b). Since HDAC2 plays an important role in regulating histone H3 and H4 acetylation, which is essential for transcription, we next analyzed the acetylation status of histones H3 and H4 on the promoters of proinflammatory genes in either LPS‐stimulated HDAC2 knockdown macrophages or control macrophages (HDAC6 knockdown and shRNA Ctrl). Similar levels of acetylated histones H3 and H4 were observed at the IL‐12p40, TNF‐α and iNOS promoter regions 60 min after stimulation (Supplementary figure 4c, d), thereby suggesting that HDAC2 does not bind directly to the promoters of proinflammatory genes and does not regulate their transcript levels by changing the acetylation status of histones H3 and H4.

**Figure 4:**
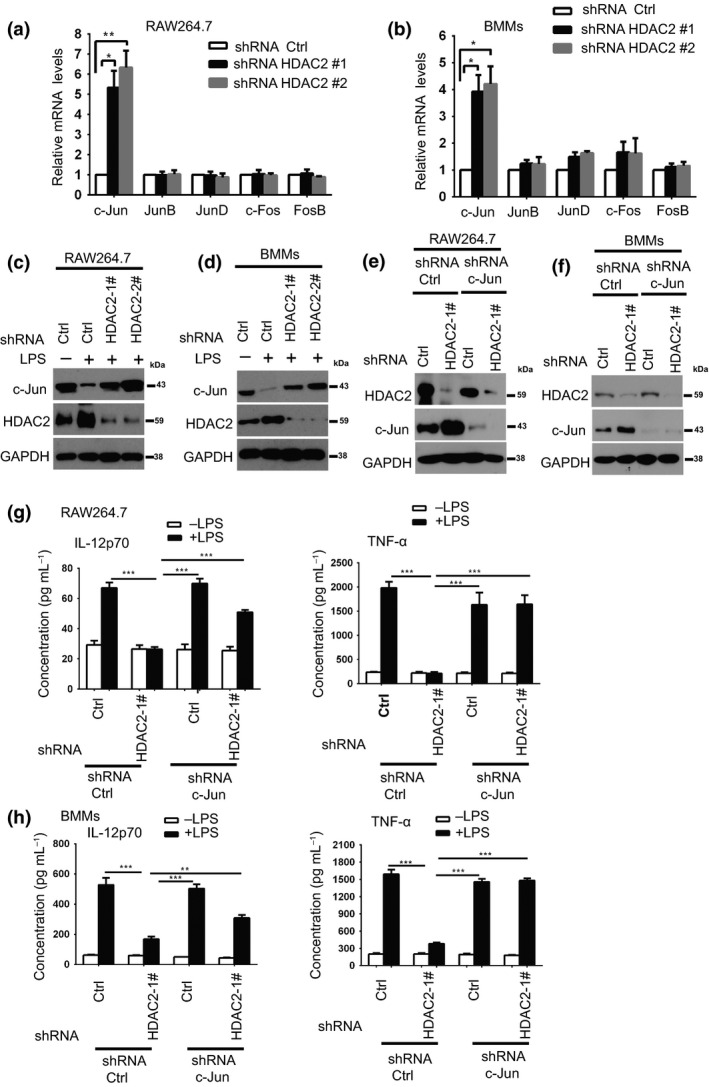
HDAC2 influences LPS‐triggered proinflammatory cytokine production by regulating c‐Jun expression. **(a**,** b)** RT‐qPCR was used to assess AP‐1 mRNA levels in shRNA Ctrl or HDAC2 knockdown (shRNA HDAC2#1 and shRNA HDAC2#2) RAW264.7 cells or BMMs that had been treated with LPS (100 ng mL ^−1^) for 1 h. The data are presented relative to shRNA Ctrl macrophages, which were set to 1. GAPDH was used as the internal control. Data are presented as the means ± s.d. of three independent experiments. *P < 0.05, **P < 0.01 (two‐tailed Student's *t*‐test). **(c**,** d)** Macrophages (RAW264.7 cells and BMMs) stably expressing Ctrl, or HDAC2 shRNAs were treated as described in **a**,** b**. Western blots were performed to assess levels of the c‐Jun protein in the lysates of Ctrl or HDAC2 shRNAs macrophages (RAW264.7 cells **(c)** and BMMs **(d)**) cultured in the presence or absence of LPS for 1 h. **(e**,** f)** Macrophages stably expressing the indicated shRNAs were lysed and cell lysates were then blotted with the indicated antibodies. Western blots were used to detect knockdown efficiency. **(g**,** h)** Macrophages (RAW264.7 cells and BMMs) stably expressing Ctrl, HDAC2 shRNA, c‐Jun shRNA or a combination of HDAC2 and c‐Jun shRNAs were treated as described in **e**,** f**. These macrophages were treated with or without 100 ng mL ^−1^ LPS for 24 h. After 24 h, the supernatants were collected to determine the IL‐12p70 and TNF‐α concentrations using ELISAs. The data are presented as the means ± s.d. of three independent experiments.*P < 0.05, **P < 0.01, and ***P < 0.001 (one‐way ANOVA).

Next, we investigated the effect of HDAC2 knockdown on LPS‐induced downstream signaling pathways in macrophages, such as NF‐κB, IRF3 and MAPK pathways.^3^ No significant differences in the expression or phosphorylation of NF‐κB, IRF3, ERK and p38 were observed between HDAC2 knockdown BMMs and shRNA Ctrl BMMs following stimulation with LPS, thereby suggesting that HDAC2 does not affect LPS‐induced activation of the NF‐κB, IRF3 and MAPK pathways. Similar results were also obtained using RAW264.7 macrophages (data not shown). AP‐1 complexes, including Jun (c‐Jun, JunB and JunD) and Fos (c‐Fos and FosB) proteins,^28^ are also important for the LPS‐triggered immune response. Thus, the expression of AP‐1 in LPS‐stimulated HDAC2 knockdown macrophages was determined. As shown in Figure 4a, b, the LPS‐mediated induction of c‐Jun gene expression was markedly increased in the HDAC2 knockdown macrophages compared with the shRNA Ctrl cells. However, the expression of other AP‐1 subunits (JunB, JunD, c‐Fos and FosB) was not significantly different between the HDAC2 knockdown and shRNA Ctrl macrophages (Figure 4a, b). Western blots confirmed that HDAC2 knockdown increased c‐Jun expression (Figure 4c,d). Furthermore, overexpression of HDAC2 decreased the level of the c‐Jun protein (Supplementary figure 2a, d).

Next, we clarified the role of c‐Jun in the LPS‐triggered HDAC2‐ proinflammatory gene signaling pathway. RAW264.7 cells or BMMs were co‐infected with the HDAC2 shRNA and c‐Jun shRNA to simultaneously silence HDAC2 and c‐Jun expression, and we examined whether c‐Jun knockdown rescued the inhibitory effect of HDAC2 knockdown on proinflammatory gene expression. The observed effect was indeed mediated by inhibition of HDAC2 and c‐Jun, as lower levels of the HDAC2 and c‐Jun mRNAs (Supplementary figure 5a) and proteins were detected in cells infected with a lentivirus carrying HDAC2 and c‐Jun‐specific shRNAs (Figure 4e, f). Notably, c‐Jun knockdown rescued the HDAC2 knockdown‐mediated alterations in LPS‐induced IL‐12p70 and TNF‐α expression (Figure 4g, h). Based on these results, HDAC2 promotes the LPS‐triggered innate immune response by regulating c‐Jun expression. Additionally, iNOS expression, which was downregulated in the HDAC2 knockdown macrophages, was not rescued by c‐Jun knockdown (Supplementary figure 6a).

### HDAC2 directly regulates the transcription of the c‐Jun gene

**Figure 5:**
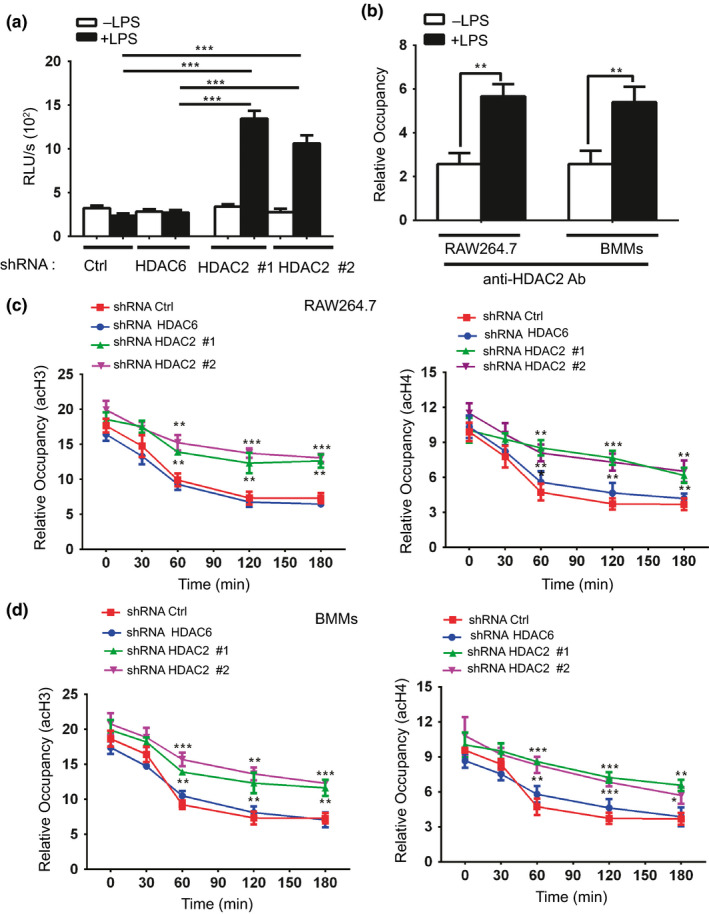
HDAC2 directly regulates c‐Jun gene transcription. **(a)** Luciferase activity was assessed in lysates of HDAC2 knockdown or control (shRNA Ctrl and shRNA HDAC6) RAW264.7 cells that had been transfected with c‐Jun luciferase reporter plasmids and stimulated with or without LPS for 24 h. The results are normalized to the Renilla luciferase activity. Data are presented as the means ± s.d. of three independent experiments (two‐way ANOVA). **(b)** A chromatin immunoprecipitation (ChIP) assay was utilized to assess binding of HDAC2 to the c‐Jun promoter in RAW264.7 cells or BMMs cultured in the presence or absence of LPS for 60 min. Immunoglobulin G (IgG) was used as an immunoprecipitation control. Data are presented as the means ± s.d. of three independent experiments. (two‐tailed Student's *t*‐test). **(c**,** d)** The c‐Jun promoter regions were assessed by chromatin immunoprecipitation (IP) of LPS‐treated control (shRNA Ctrl and shRNA HDAC6) or HDAC2 knockdown macrophages that were incubated with a control IgG antibody or with an antibody against acetylated histone 3 (acH3) and acetylated histone 4 (acH4) for 30 min, 1, 2 or 3 h, followed by quantitative PCR analysis. Relative occupancy values generated by the quantitative PCR analysis were normalized to control IgG precipitations. Data are presented as the means ± s.d. of three independent experiments (two‐way ANOVA). *P < 0.05, **P < 0.01, and ***P < 0.001 compared with the shRNA Ctrl.

We transfected HDAC2 knockdown or control (HDAC6 knockdown and shRNA Ctrl) RAW264.7 cells with a reporter gene containing the c‐Jun promoter fused to a luciferase gene to determine whether the function of HDAC2 was required for differential c‐Jun expression. Subsequently, cells were unstimulated or stimulated with LPS. As shown in Figure 5a, the LPS‐stimulated control RAW264.7 cells exhibited significantly less luciferase activity than the HDAC2 knockdown RAW264.7 cells. These findings confirmed that HDAC2 directly targets the c‐Jun promoter.

In addition, ChIP assays were performed to further investigate the interaction of HDAC2 with the c‐Jun promoter in macrophages. LPS induced the binding of HDAC2 to the c‐Jun promoter region (Figure 5b), and this interaction was sustained for up to 3 h after stimulation (data not shown). The ChIP analysis of the acetylation status of histones H3 and H4 at the c‐Jun promoter region showed that acetylation of histones H3 and H4 was dramatically decreased in the shRNA Ctrl macrophages by 60 min after LPS stimulation (Figure 5c, d). In contrast, histone H3 and H4 acetylation at the promoter region of c‐Jun in the HDAC2 knockdown macrophages was sustained at a high level for 3 h in response to LPS stimulation (Figure 5c, d). Thus, LPS stimulation enhances the recruitment of HDAC2 to the c‐Jun promoter and subsequently suppresses the acetylation of histones H3 and H4 at the c‐Jun promoter.

### HDAC2 inhibits the formation of c‐Jun/c‐Jun homodimers and the nuclear receptor corepressor (NCoR) complex in macrophages

**Figure 6:**
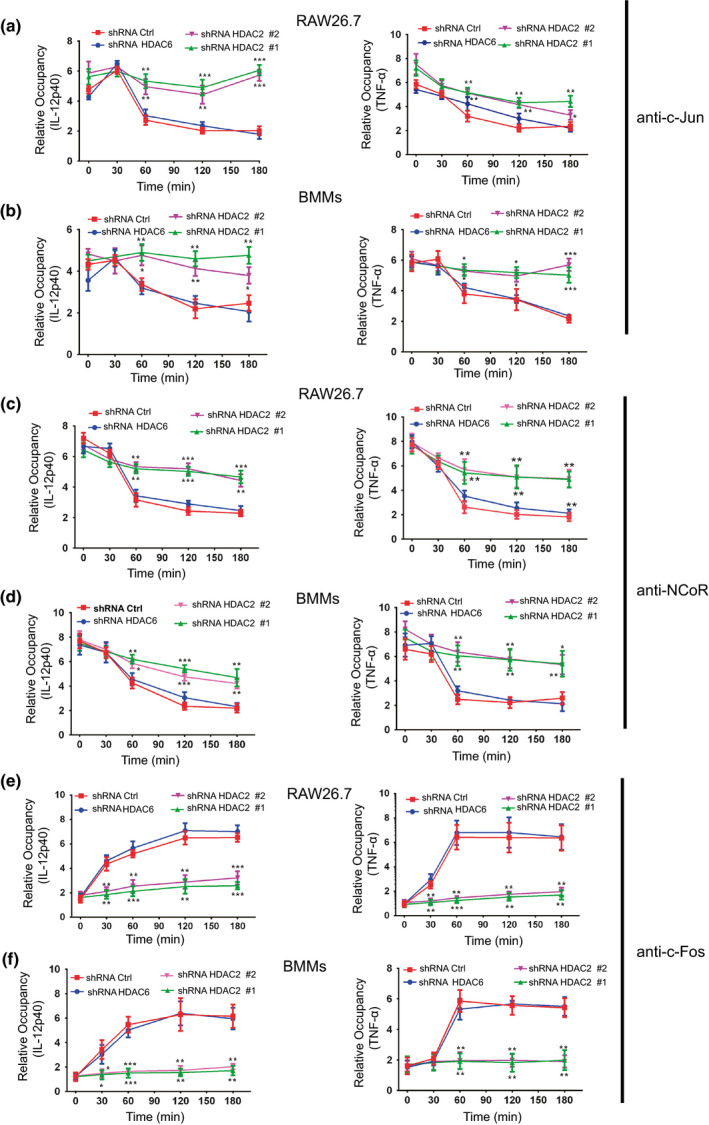
HDAC2 inhibits the formation of c‐Jun/c‐Jun homodimers and nuclear receptor corepressor (NCoR) complexes in macrophages. A chromatin immunoprecipitation (ChIP) assay was utilized to detect the binding of c‐Jun **(a**,** b)**, NCoR **(c**,** d)** and c‐Fos **(e**,** f)** to the IL‐12p40 and TNF‐α promoters in control (shRNA Ctrl and shRNA HDAC6) or HDAC2 knockdown macrophages that had been stimulated with LPS for 30 min, 1, 2 or 3 h. These levels were assessed with two independent primers for each promoter. Immunoglobulin G was used as an immunoprecipitation control. Relative occupancy values generated by quantitative PCR analysis were normalized to control IgG precipitations. Data are presented as the means ± s.d. of three independent experiments. *P < 0.05, **P < 0.01, and ***P < 0.001 compared with the shRNA Ctrl (two‐way ANOVA).

AP‐1/c‐Jun is activated by JNK and directs the exchange of c‐Jun for c‐Jun/c‐Fos heterodimers, thereby resulting in the loss of NCoR complexes from AP‐1 target proinflammatory genes. C‐Jun/c‐Jun homodimers and NCoR comprise NCoR corepressor complexes that bind to the promoters of proinflammatory genes to suppress gene transcription. LPS stimulation results in the exchange of c‐Jun for c‐Fos and subsequent NCoR clearance from the promoters of proinflammatory genes.^29^ ChIP experiments were conducted in LPS‐treated control (HDAC6 knockdown and shRNA Ctrl) macrophages and HDAC2 knockdown macrophages to detect the binding of c‐Jun, NCoR and c‐Fos to the promoter of proinflammatory genes to examine the possibility that HDAC2 knockdown blocks the exchange of c‐Jun for c‐Fos and enhances c‐Jun/c‐Jun NCoR corepressor complex formation at the promoters of proinflammatory genes after LPS treatment in macrophages. As shown in Figure 6a–d, the basal levels of binding of NCoR and c‐Jun to the promoters of the IL‐12p40 and TNF‐α genes were not different between HDAC2 knockdown and control macrophages. After LPS stimulation, the binding of NCoR and c‐Jun to the IL‐12p40 and TNF‐α promoters peaked at 30 min, followed by a rapid decrease of control macrophages at 1 h. However, the binding of NCoR and c‐Jun to the IL‐12p40 and TNF‐α promoters in the HDAC2 knockdown macrophages was sustained for 3 h at a high level in response to LPS stimulation (Figure 6a–d). Intriguingly, ChIP experiments that investigated the TNF‐α and IL‐12p40 promoters in control and HDAC2 knockdown macrophages revealed an obvious increase in the occupancy of c‐Fos in control cells that peaked at 2 h (Figure 6e, f). Therefore, HDAC2 plays a vital role in blocking the formation of the c‐Jun‐NCoR complex at the promoters of proinflammatory genes in macrophages following stimulation with LPS, which promotes proinflammatory gene expression. Based on these results, we proposed a working model in which HDAC2 mediates the LPS‐triggered inflammatory response by directly binding to the c‐Jun promoter (Supplementary figure 7).

## Discussion

LPS, a classic pathogen‐associated molecular pattern, dynamically regulates innate immune responses and contributes to pathology during gram‐negative bacteria‐induced sepsis. TLR4, which activates signal transduction pathways, is involved in the LPS– or bacterium–host interactions.^2^ Although various signaling molecules are thought to be involved in the mechanism regulated by the LPS‐TLR4 pathway, epigenetic control of the LPS‐triggered innate immune response remains unclear and is currently under extensive investigation.

HDACs are important epigenetic regulators that control chromatin structure, transcription factor DNA accessibility, and gene expression by changing the histone acetylation status at gene promoters.^20^ In this experiment, our findings identified the HDAC family members that contribute to this process, and more specifically, we identified HDAC2 as an important regulator of LPS‐triggered innate immunity in macrophages. Knockdown of HDAC2 suppressed proinflammatory gene expression and altered the secreted cytokine profile. HDAC2 knockdown BMMs compromised LPS‐induced endotoxic shock *in vivo* and protected mice from lethal challenge with LPS and live gram‐negative bacteria. We also identified a negative role for HDAC2 in regulating c‐Jun expression. Notably, c‐Jun was identified as a critical factor by which HDAC2 regulates the LPS‐induced inflammatory response after LPS treatment.

HDAC2, a member of the class I HDAC family, was reported to play vital roles in early synaptogenesis and cancer development.^30^, ^31^ The roles of class I HDACs in regulating proinflammatory gene expression remain controversial. Class I HDACs have been reported to exert negative effects on the regulation of inflammatory responses in macrophages.^32^, ^33^ Nevertheless, in our study, LPS‐induced HDAC2 expression in macrophages exerted a positive effect on the induction of proinflammatory gene expression. HDACs promote and repress the transcription of genes by adding and removing acetyl groups from the lysine residues of histone proteins. Because the proinflammatory genes are divergent and the regulation of the histone proteins at the promoters of the diverse proinflammatory genes is complicated, the role of HDAC2 in regulating the acetylation of these histone proteins at the promoters of diverse proinflammatory genes varies.

According to Zhang *et al*., Tet2 recruits HDAC2 to the IL‐6 promoter and represses IL‐6 transcription via histone deacetylation.^34^ However, in the current study, HDAC2 did not directly bind to the promoters of proinflammatory genes or regulate their transcription in response to LPS stimulation. Furthermore, the translation of the NF‐κB, IRF3, ERK and p38 proteins that play critical roles in regulating the levels of proinflammatory genes was not affected by HDAC2. Rather, HDAC2 directly interacted with the c‐Jun promoter and participated in silencing c‐Jun expression.

The activity of the c‐Jun protein is important for regulating the transcription of a series of inflammatory pathway genes.^35^, ^36^ Specific deletion of c‐Jun in epithelial tissues can lead to a psoriasis‐like phenotype in mice. Both the constitutive deletion of c‐Jun and the conditional knockout of c‐Jun in epithelial tissues increased the levels of some of the proinflammatory factors that are involved in psoriasis, such as TNF‐α and IL‐6.^37^ C‐Jun is one of the AP‐1 subunits. Different AP‐1 subunits dimerize in various combinations and bind the promoters and enhancers of target genes to regulate their expression. Activated JNK phosphorylates the N terminus of c‐Jun bound to target gene promoters, resulting in an exchange of c‐Jun dimers for c‐Fos heterodimers. This process induces the transactivation of a set of inflammatory pathway genes.^37^ Consistent with this interpretation, c‐Jun is upregulated in LPS‐stimulated HDAC2 knockdown macrophages, resulting in increased binding of c‐Jun to specific gene promoters and the inhibition of c‐Fos binding to DNA. Thus, in the absence of HDAC2, upregulation of c‐Jun may enhance the DNA binding ability of c‐Jun/c‐Jun homodimers and inhibit the exchange of c‐Jun dimers for c‐Jun/c‐Fos heterodimers, enabling c‐Jun/c‐Jun homodimers and NCoR to form NCoR corepressor complexes and bind the promoters of proinflammatory genes, thereby suppressing gene transcription.^27^, ^35^

HDAC2 was required for the LPS‐stimulated inflammatory response in macrophages in the present study. HDAC2 knockdown inhibited proinflammatory gene expression and protected mice from endotoxic shock following stimulation with LPS. Mechanistically, HDAC2 specifically bound to the promoter of the c‐Jun gene and inhibited its transcription. This process repressed the formation of NCoR corepressor complexes at the promoter regions of proinflammatory genes, thereby enhancing their expression, which ultimately enhanced the LPS‐induced innate immune response. Therefore, strategies designed to regulate HDAC2 expression in macrophages should be exploited to regulate macrophage function and the subsequent effects on inflammation.

## Methods

### Mice, cells and LPS

We used 6‐ to 8‐week‐old C57BL/6 female mice. We purchased RAW264.7 and HEK293T cells from ATCC. RAW264.7 mouse macrophage cells and HEK293T cells were cultured in RPMI 1640 medium and DMEM, respectively, supplemented with 10% FBS. BMMs (bone‐marrow‐derived macrophages) were obtained from femoral and tibial bone marrow suspensions and plated in 10‐cm plates at a density of 1 × 10^6^ cells. Additionally, these cells were differentiated in 10 mL of BMM medium (RPMI1640 containing 25 mm HEPES, 10% low endotoxin FBS, 10 ng mL^−1^ M‐CSF (Sigma, SRP3221), 2 mm L‐glutamine and 1 mm gentamycin) for 6 days. Stimulations and transfection were performed on day 8. LPS (100 ng mL^−1^) from the *Escherichia coli* serotype 0111B4 was purchased from Sigma‐Aldrich (St Louis, MO, USA).

### Gene knockdown and virus infection

For gene knockdown in macrophages, the corresponding shRNAs and non‐targeting control shRNA (Ctrl) were cloned into pLKO.1 (Addgene plasmid # 10878) or pLKO‐Tet‐On cloning vectors.^38^ The sequences of all plasmids were verified by DNA sequencing. Primers used for shRNAs are listed in Supplementary table 1. For knockdown lentivirus packaging, foreign DNA, the packaging plasmid, psPAX2, and envelope plasmid pMD2.G were transfected together at a 4:3:1 ratio into HEK293T cells using the Lipofectamine 2000 transfection reagent (Invitrogen) according to the manufacturer's recommendations.

Culture supernatants were collected and centrifuged at 800 *g* 48 h after transfection. Supernatants and 8 μg mL^−1^ polybrene, which enhanced the infection efficiency, were used to infect the RAW264.7 cells or bone‐marrow‐derived macrophages (BMMs) for 24 h. Twenty‐four hours after the infection, the medium was replaced and cells were cultured for an additional 24 h. Puromycin was added to the media at a concentration of 2 μg mL^−1^ to select the infected cells. Finally, media were replaced with fresh puromycin‐containing media as needed every few days until cells stably expressing the target shRNA were selected.

### ELISA

ELISAs were performed as previously described.^25^ Briefly, culture media were collected and centrifuged at 1000*g* in a refrigerated centrifuge for 5 min at 4°C and examined for the presence of mouse IL‐12p70 and TNF‐α (Shanghai ExCell Biology, Inc., Shanghai, China). Assays were performed according to the manufacturer's protocol, and the absorbance at 450 nm was recorded using a microplate reader (Bio‐Rad, Hercules, CA, USA). The conditioned culture media were collected and centrifuged at 1000*g* in a refrigerated centrifuge for 5 min at 4°C. Nitrite levels in the supernatant were measured using the Griess assay.^39^

### Real‐time quantitative PCR

Real‐time quantitative PCR (RT‐qPCR) was performed using previously described methods.^25^ Briefly, RNA was isolated with TRIzol‐A^+^ (Tiangen, Shanghai, China), and reverse transcriptase reaction kit (Tiangen, Shanghai, China) was used to transcribe cDNAs from 1000 ng of total RNA according to the manufacturer's instructions. SYBR Green Real‐time PCR Master Mix (Bio‐Rad) and the Stratagene Mx3000 QPCR system (Stratagene, Agilent Technologies, Palo Alto, CA, USA) were used for RT‐qPCR. The results were normalized to the GAPDH gene. The relative level of each mRNA was quantified using the 2^−▵▵^Ct method.^40^ All primers used for RT‐qPCR are listed in Supplementary table 2.

### Chromatin immunoprecipitation (ChIP) assays

ChIP assays were performed as previously described.^27^, ^41^, ^42^ Specific antibodies were used to immunoprecipitate the chromatin‐associated proteins, after which the DNA was purified, suspended in TE buffer and analyzed using SYBR Green fluorescence dye. The primers used for ChIP‐qPCR are listed in Supplementary table 3. The antibodies used in the ChIP assay are listed in Supplementary table 4.

### Western blot

Western blotting was used to determine protein levels using previously described methods.^41^, ^42^ Briefly, cells were harvested and lysed in lysis buffer containing protease inhibitors. Twenty micrograms of protein were resolved on SDS‐PAGE gels and then transferred to PVDF membranes (Perkin Elmer Life Sciences, Waltham, MA, USA). Then the membranes were sequentially exposed to specific primary antibodies. GAPDH was used as a loading control. After incubation with the HRP‐conjugated secondary antibodies, signals were visualized using enhanced chemiluminescence (ECL) (ImageQuant LAS 4000 mini, General Electric Company, Fairfield Municipal, CT, USA). The antibodies used are listed in Supplementary table 4.

### Reporter gene assays

A c‐Jun gene promoter‐based reporter construct plasmid was generated by PCR amplification of the −560/+2 fragment of the c‐Jun promoter and cloning of the resulting fragment into a *Hin*dIII/*Xho*I‐digested pGL2. The resulting construct was designated as pGL2‐c‐Junpro. The RAW264.7 cell line transduced with lentivirus carrying scrambled shRNA (Ctrl), HDAC6 or HDAC2 shRNAs was co‐transfected with a mixture of the pGL2‐c‐Junpro plasmid and pRL‐TK‐Renilla‐luciferase plasmid. Twenty‐four hours after transfection, RAW264.7 cells were stimulated with LPS. We used a luciferase assay system (Promega, Madison, WI, USA) to detect the luciferase activity in the cells. We normalized all the data and divided the firefly luciferase activity levels by the Renilla luciferase activity levels.

### BMM adoptive transfer, LPS challenge and bacterial sepsis model

We intravenously injected C57BL/6 mice with GdCl_3_ (Sigma‐Aldrich, 15 mg kg^−1^) as previously described^26^ to eliminate macrophages *in vivo* before the mice received an intravenous injection of HDAC2 knockdown or control BMMs (1 × 10^7^). After 24 h, we intraperitoneally injected these mice with LPS (5 mg kg^−1^) and measured the serum levels of TNF‐α and IL‐12p70 using ELISAs. In another model, LPS (15 mg kg^−1^) was intraperitoneally injected into mice to induce endotoxic shock, after which the survival rate of the mice was measured every 12 h. For the bacterial infection, we collected the *E. coli* serotype 0111:B4 cells in midlogarithmic growth counted them on agar plates and then resuspended them in PBS. We intraperitoneally injected the mice with approximately 1 × 10^7^ *E. coli*.

### Statistical analysis

Data are presented as the means± standard deviation (s.d.) from at least three independent experiments. Statistical analyses were performed using one‐way ANOVA, two‐way ANOVA, Student's *t‐*test (two‐tailed unpaired) and the log‐rank (Mantel–Cox) test. Statistical significance is presented as follows: n.s., not significant; *P <* *0.05; **P < 0.01 and ***P < 0.001.
