# Supplementary material for: Histone deacetylase 2 is essential for LPS‐induced inflammatory responses in macrophages
Source: Immunol Cell Biol. 2018 Oct 31;97(1):72–84. doi: 10.1111/imcb.12203 (PMC7379312; doi:10.1111/imcb.12203)
Supplement: Supplementary file 1 [file IMCB-97-72-s001.pdf]

SUPPLEMENTARY INFORMATION

Histone deacetylase 2 is essential for LPS-induced inflammatory responses in macrophages

Chenming Wu, Ang Li, Jian Hu, Jiuhong Kang

Supplementary methods

Cell survival assays

Infected cells (2×10^3 cells/well) were seeded into 96-well culture plates. Cell proliferation was detected at days 1, 2, 3, 4, 5 and 6 using tetrazolium compound based CellTiter 96® AQueous One Solution Cell Proliferation (MTS) assay (Promega). We added 15μL MTS reagents to each well and incubated the cells for 2-4 hours at 37 ℃. Then, the plates were read at 490 nm in an EPOCH2 microplate reader (BioTek instruments).

Gene overexpression

For gene overexpression, the complementary DNAs were inserted into 3*FLAG-pLVX3 lentivirus constructs. All the plasmids were verified by DNA sequencing. For overexpression lentivirus packaging, foreign DNA, the packaging plasmid, psPAX2, and envelope plasmid pMD2.G were transfected together at a 4:3:1 ratio into HEK293T cells using the Lipofectamine 2000 transfection reagent (Invitrogen) according to the manufacturer's recommendations. Culture supernatants were collected and centrifuged at 2,000 rpm 48 hours after transfection. Supernatants and 8 μg mL-1 polybrene, which enhanced the infection efficiency, were used to infect the RAW264.7 cells or bone marrow-derived macrophages (BMMs) for

24 h. Twenty-four hours after the infection, the medium was replaced and cells were cultured for an additional 24 h. Puromycin was added to the media at a concentration of 2 μg mL-1 to select the infected cells. Finally, media were replaced with fresh puromycin-containing media as needed every few days until cells stably expressing the target genes were selected.

Fuorescent-activated cell sorter (FACS) analysis

FACS analysis was performed as previously described.[1,2] The antibodies are listed in Supplementary table 4.

Supplementary Figure Legends

Supplementary Figure 1. HDAC2 knockdown does not affect cell viability

(a) Macrophages (RAW264.7 cells and BMMs) stably expressing scrambled short hairpin RNA (shRNA Ctrl), or two different HDAC2-specific shRNAs (shRNA HDAC2 #1 and shRNA HDAC2 #2) lentivirus were lysed and cell lysates were blotted with the indicated antibodies. **(b-c)** Cells from **(a)** were measured for **(b-c)** cell proliferation. Data are presented as the means ± SD (n=6) for cell proliferation assays. n.s.: not significant. Data compared with the shRNA Ctrl. (two-tailed Student's *t*-test)

Supplementary Figure 2. HDAC2 overexpression increases the expression of proinflammatory genes in macrophages

(a) RAW264.7 macrophages stably expressing Flag-HDAC2 lentivirus (HDAC2-OV) or empty vector (Vector) were lysed and cell lysates were blotted with the indicated

antibodies. **(b)** The cells from **(a)** were treated with 100 ng mL^{-1} LPS for 6 h. Levels of the IL-12p40, TNF-α and iNOS mRNAs were determined using RT-qPCR. **(c)** The cells from **(a)** were treated with 100 ng mL^{-1} LPS for 24 h. After 24 h, the supernatants were collected to determine the IL-12p70 and TNF-α concentrations using ELISAs. Additionally, nitrite concentrations, a stable end product of NO production, were detected using Griess reagent. **(d)** BMMs stably expressing Flag-HDAC2 lentivirus (HDAC2-OV) or empty vector (Vector) were lysed and cell lysates were blotted with the indicated antibodies. **(e)** The cells from **(d)** were treated with 100 ng mL^{-1} LPS for 6 h. Levels of the IL-12p40, TNF-α and iNOS mRNAs were determined using RT-qPCR. **(f)** The cells from **(d)** were treated with 100 ng mL^{-1} LPS for 24 h. After 24 h, the supernatants were collected to determine the IL-12p70 and TNF-α concentrations using ELISAs. Additionally, nitrite concentrations, a stable end product of NO production, were detected using Griess reagent. The data shown in **(b)** and **(e)** are presented relative to Vector-macrophages, which set to 1. GAPDH was used as the internal control. The data shown in **(b), (c), (e)** and (**f**) are means ± SD of three independent experiments.**P* < 0.05, ***P* < 0.01 compared with the Vector macrophages (two-tailed Student's *t*-test).

Supplementary Figure 3. $GdCl_3$ selectively depletes circulating mononuclear cells of the monocyte/macrophage lineage

(a) Representative FACS data and averaged percentages of the circulating macrophages in mice treated GdCl3. ****P < 0.0001 (two-tailed Student's *t*-test)
Data are expressed as means ± SD of three independent experiments.

Supplementary Figure 4. HDAC2 indirectly influences the expression of TLR4-triggered proinflammatory genes

(a-b) HDAC2 binding to the IL-12p40, TNF-α and iNOS promoters was detected with the ChIP assay. These assays were conducted with a control immunoglobulin G

(IgG) antibody or with an HDAC2 antibody in RAW 264.7 cells or BMMs with or without LPS treatment, followed by quantitative PCR analysis of the IL-12p40, TNF-α and iNOS promoter region. Data shown are means ± SD of three independent experiments. **(c-d)** Chromatin immunoprecipitation (IP) of LPS-treated control (shRNA Ctrl and shRNA HDAC6) or HDAC2 knockdown macrophages with a control IgG antibody or with an antibody to acH3 or acH4, followed by quantitative PCR analysis of the IL-12p40, TNF-α and iNOS promoter region. Relative occupancy values generated by quantitative PCR analysis were normalized to control IgG precipitations. Data shown are means ± SD of three independent experiments

Supplementary Figure 5. The knockdown efficiency of HDAC2 shRNA and c-Jun shRNA on mRNA level

(a) Macrophages (RAW264.7 cells and BMMs) stably expressing Ctrl, HDAC2,c-Jun, combination of HDAC2 and c-Jun shRNAs were extracted for detecting the mRNA levels of the targeting genes (c-Jun and HDAC2) using RT-qPCR. RT-qPCR was utilized to detect the knockdown efficiency. The data are presented relative to shRNA Ctrl-macrophages, which set to 1. GAPDH was used as the internal control. Data shown are means ± SD of three independent experiments. $^{**}P < 0.01$, $^{***}P < 0.001$ compared with the shRNA Ctrl (two-tailed Student's *t*-test).

Supplementary Figure 6. iNOS expression, downregulated in the HDAC2 knockdown macrophages, cannot be rescued by c-Jun knockdown

(a) Macrophages (RAW264.7 cells and BMMs) stably expressing Ctrl, HDAC2 shRNA,c-Jun shRNA or a combination of HDAC2 and c-Jun shRNAs were from Figure 4e-4f. These macrophages were treated with or without 100 ng mL^{-1} LPS for

24 h. After 24 h, the supernatants were collected and nitrite concentrations, a stable end product of NO production, were detected using Griess reagent.

Supplementary Figure 7. A working model of LPS-induced inflammatory response mediated by HDAC2

Supplementary table 1: Primer sets used for shRNA

Gene symbols	Sequence (5' to 3')<br>target sequence	Applications
shRNA<br>Ctrl	CCTAAGGTTAAGTCGCCCTCG	vector
shRNA<br>HDAC6	TGAGGATGACCCTAGTGTATT	vector
shRNA<br>HDAC2 #1	ATGTTGGACATATGAGACTGCAGTT	vector
sh RNA<br>HDAC2 #2	GTATCATCAGAGAGTCTTATT	vector
shRNA<br>c-Jun	AGGGAACAGGTGGCACAGCTT	vector

Supplementary table 2: Primer sets used in RT-qPCR assays

Gene symbols	Primer sequences (5' to 3')

HDAC1	GGGCACCAAGAGGAAAGTCT	Sense
	AGCAAATTGTGAGTCATGCG	Antisense
HDAC2	ATGGCGTACAGTCAAGGAGG	Sense
	TGCGGATTCTATGAGGCTTCA	Antisense
HDAC3	AGTCAGCCCCACCAATATGC	Sense
	CCTGTGTAACGGGAGCAGAACT	Antisense
HDAC8	TTTGAGCGTATTCTCTACGTGGA	Sense
	ACACTGTAGTACCGTCCCTTC	Antisense
IL-12p40	TGGTTTGCCATCGTTTTGCTG	Sense
	ACAGGTGAGGTTCACTGTTTCT	Antisense
TNF-α	GATCGGTCCCCAAAGGGAT	Sense
	ACTTGGTGGTTTGCTACGACG	Antisense
iNOS	TGGAGCGAGTTGTGGATTG	Sense
	CGTAATGTCCAGGAAGTAGGTG	Antisense
c-Jun	GAAAAGTAGCCCCCAACCTC	Sense
	AATCAGACAGGGGACACAGC	Antisense
c-Fos	TCCTTACGGACTCCCCAC	Sense
	CTCCGTTTCTCTTCCTCTTCAG	Antisense
JunB	AACTCCTGAAACCCACCTTG	Sense
	GATCCCTGACCCGAAAAGTAG	Antisense
FosB	GAAGAGACACTTACCCCAGAAG	Sense

	GCCTTTTCCTCTTCAAGCTG	Antisense
JunD	CAAGCTGGAGCGTATCTCG	Sense
	CGTGGCTGAGGACTTTCTG	Antisense
HDAC6	TGCCCACCTAACCCATTTG	Sense
	AAGCACTGATTCCCTTAGCC	Antisense
GAPDH	GTGTTCCTACCCCCAATGTGT	Sense
	ATTGTCATACCAGGAAATGAGCTT	Antisense

Supplementary table 3: Primer sets used in ChIP analysis

Gene symbols	Primer sequences (5' to 3')	
IL-12p40 promoter	GAAAACATGGGGAAAGGTGG	Sense
	ATAGAGGCGGCAATGGCTAA	Antisense
TNF-α promoter	AGAAATCAA AAGGAAATAGACAC	Sense
	ACCCTGAGAACTGAAACCCA	Antisense
iNOS promoter	TTGGCACCATCTA ACCTCAC	Sense
	TTCAAAACTGGGACTCACTA	Antisense
c-Jun promoter	CCTCCCC TGCTTTCTGGATC	Sense
	CGTCTTGGTTTGGCTGTCTA	Antisense

Supplementary table 4: List of antibodies used

Antibody target	Vendor	Catologue number	Application

HDAC1	Cell Signaling Technology, Inc	2062	Western
HDAC2	Santa Cruz	Sc-7899	Western
HDAC3	BD Transduction Laboratories	611124	Western
HDAC8	abcam	Ab39664	Western
GAPDH	abcam	Ab22556	Western
HDAC6	Proteintech	12834-1-AP	Western
c-Jun	Sigma	SAB4501604	Western
FLAG	Sigma	F1804	Western
HDAC2	Santa Cruz	sc-6296	ChIP
Ace-histone H3	Millipore	07-355	ChIP
Ace-histone H4	Millipore	07-329	ChIP
c-Jun	Cell Signaling Technology, Inc	9165	ChIP
c-Fos	Cell Signaling Technology, Inc	2250	ChIP
Nuclear Receptor Corepressor (NCoR)	abcam	ab24552	ChIP
goat IgG	Santa Cruz	Sc-2028	ChIP
mouse IgG	Millipore	MABC002	ChIP
CD11b	eBioscience	11-0112-81	FACS
F4/80	eBioscience	17-4801-80	FACS

REFERENCES

1. Li A, Chen P, Leng Y, *et al.* Histone deacetylase 6 regulates the immunosuppressive properties of cancer-associated fibroblasts in breast cancer through the STAT3-COX2-dependent pathway. *Oncogene* 2018 Jul 6. doi: 10.1038/s41388-018-0379-9.

2. Guerriero JL,Sotayo A,Ponichtera HE, *et al*. Class IIa HDAC inhibition reduces breast tumours and metastases through anti-tumour macrophages. *Nature* 2017; **543:**428-432.

Supplementary Figure 1

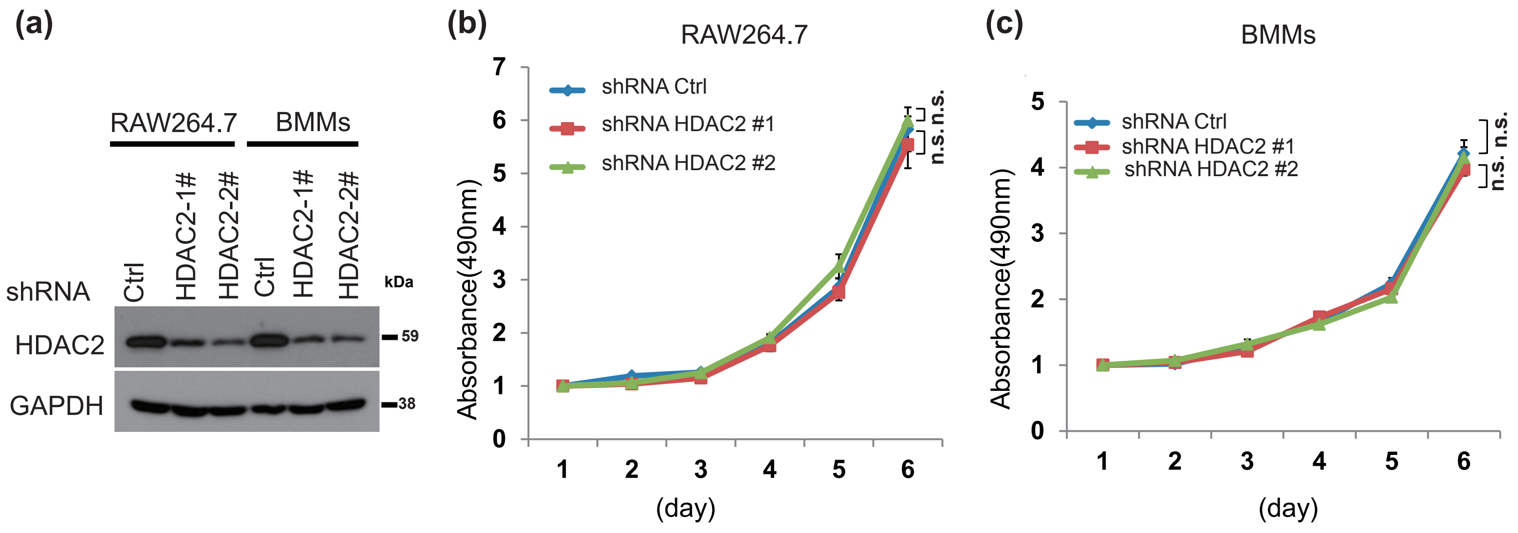

Supplementary Figure 2

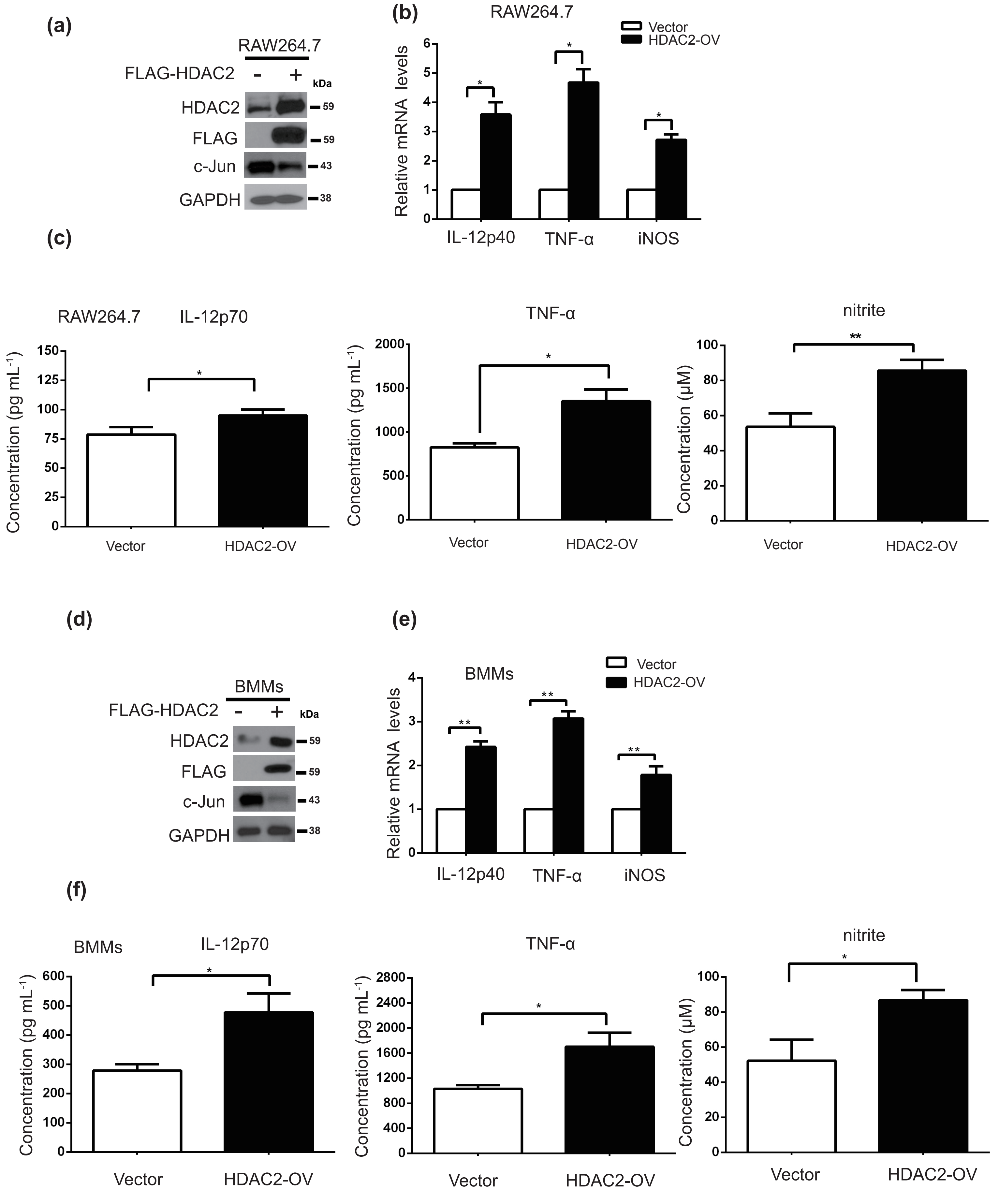

Supplementary Figure 3

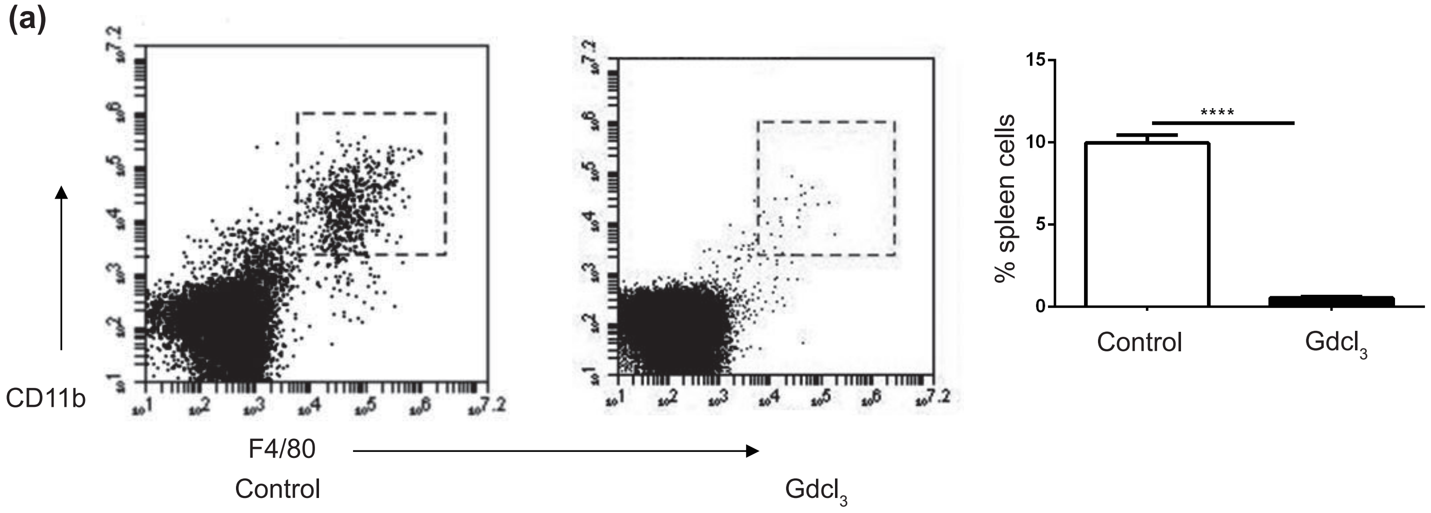

Supplementary Figure 4

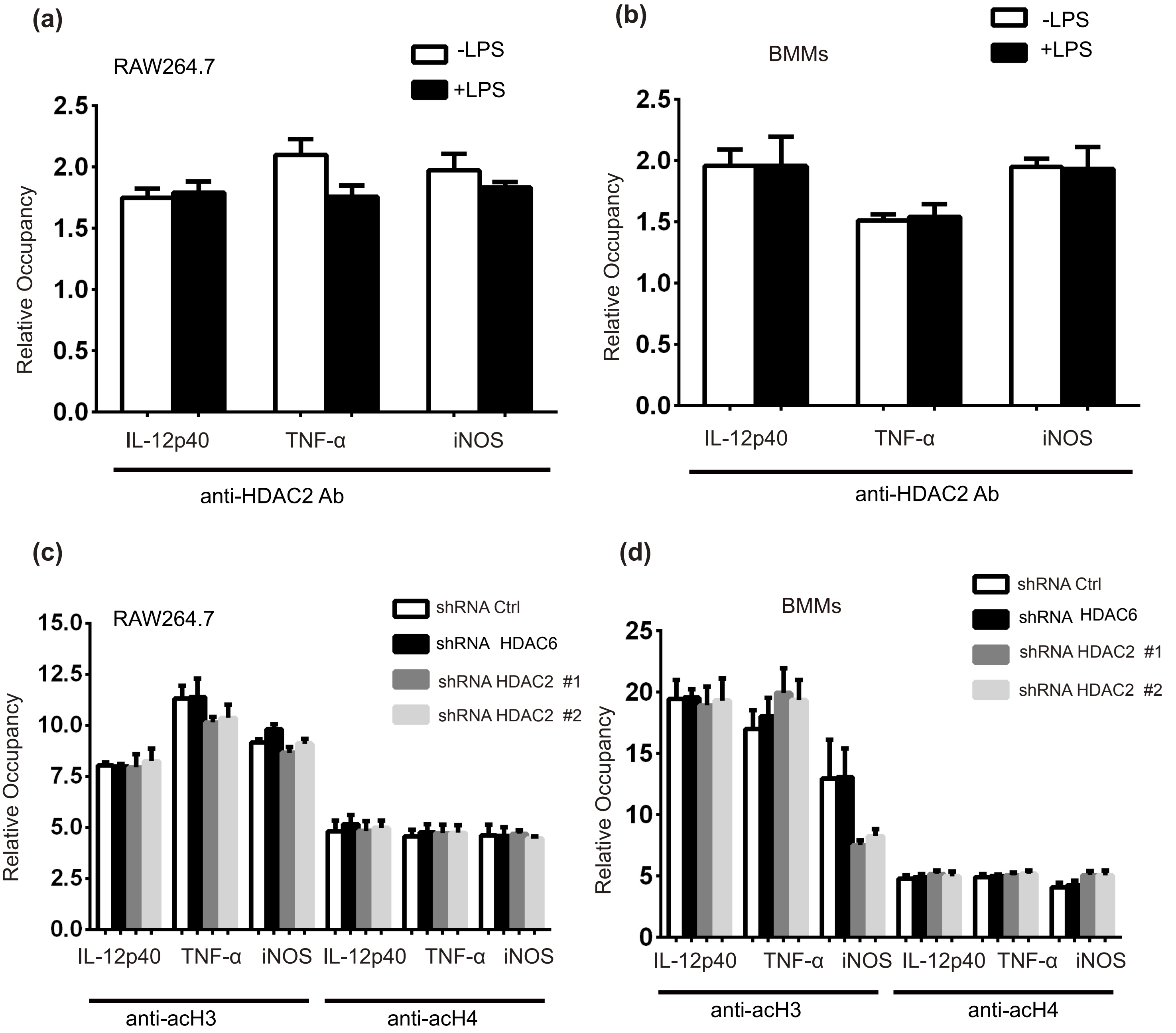

Supplementary Figure 5

(a)

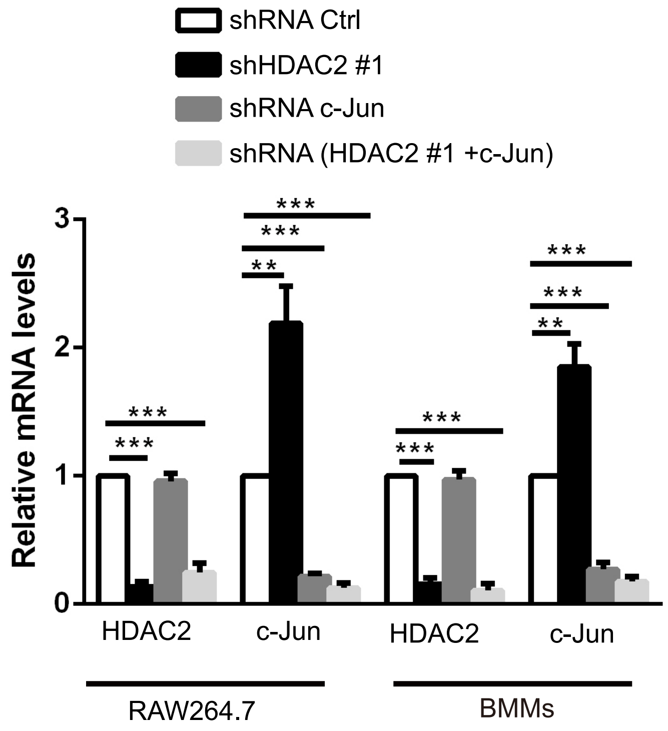

Supplementary Figure 6

(a)

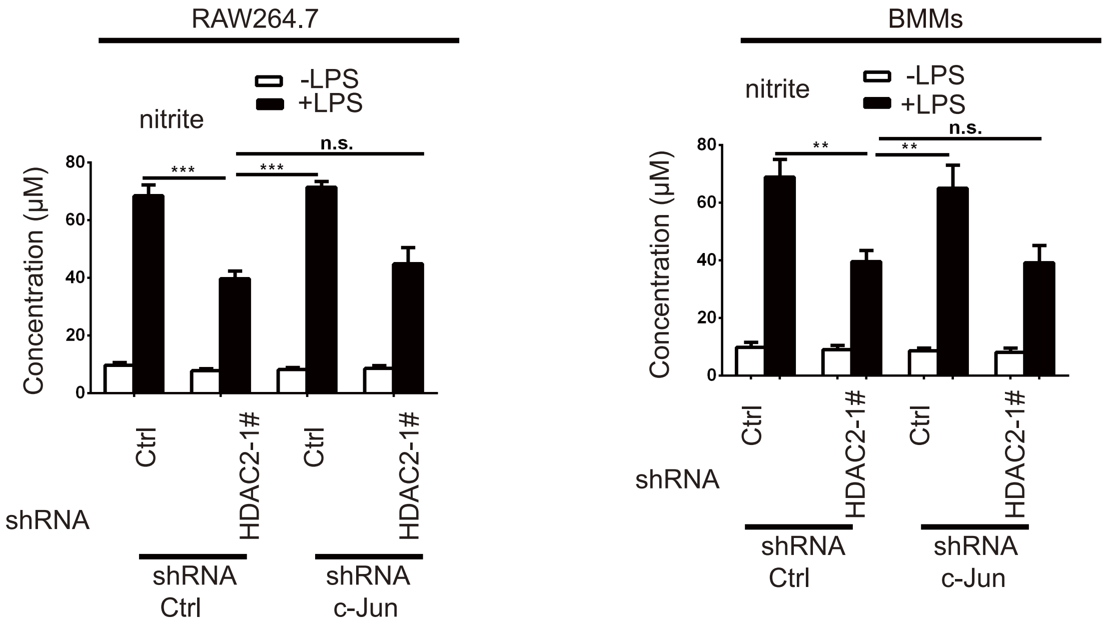

Supplementary Figure 7

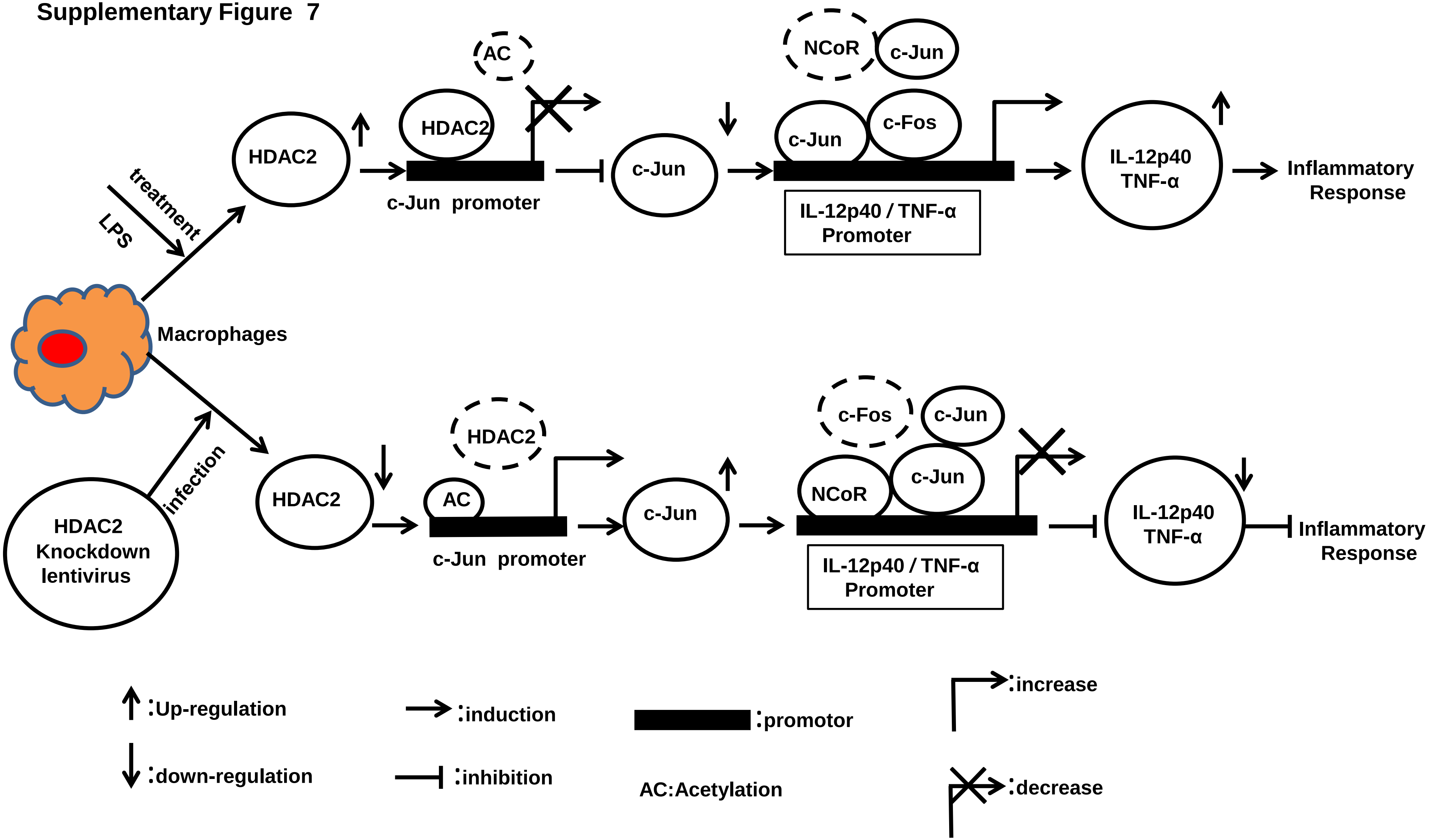

A working model of LPS-induced inflammatory response mediated by HDAC2
